# Correction to **‘**long non-coding RNAs involved in *Drosophila* development and regeneration’

**DOI:** 10.1093/nargab/lqae127

**Published:** 2024-09-14

This is a correction to: Carlos Camilleri-Robles, Raziel Amador, Marcel Tiebe, Aurelio A Teleman, Florenci Serras, Roderic Guigó, Montserrat Corominas, Long non-coding RNAs involved in *Drosophila* development and regeneration, *NAR Genomics and Bioinformatics*, Volume 6, Issue 3, September 2024, lqae091, https://doi.org/10.1093/nargab/lqae091

In the originally published version of this manuscript, an error was present in the link to the Catalan version of the abstract of the article, given at the end of the Introduction section.

The link has been corrected as follows: https://doi.org/10.5281/zenodo.13258939.

