# Supplementary material for: Long non-coding RNAs involved in Drosophila development and regeneration
Source: NAR Genom Bioinform. 2024 Aug 16;6(3):lqae091. doi: 10.1093/nargab/lqae091 (PMC11327875; doi:10.1093/nargab/lqae091)
Supplement: lqae091_Supplemental_Files [file lqae091_supplemental_files.zip › Supplementary_Figures_S1_S7.pdf]

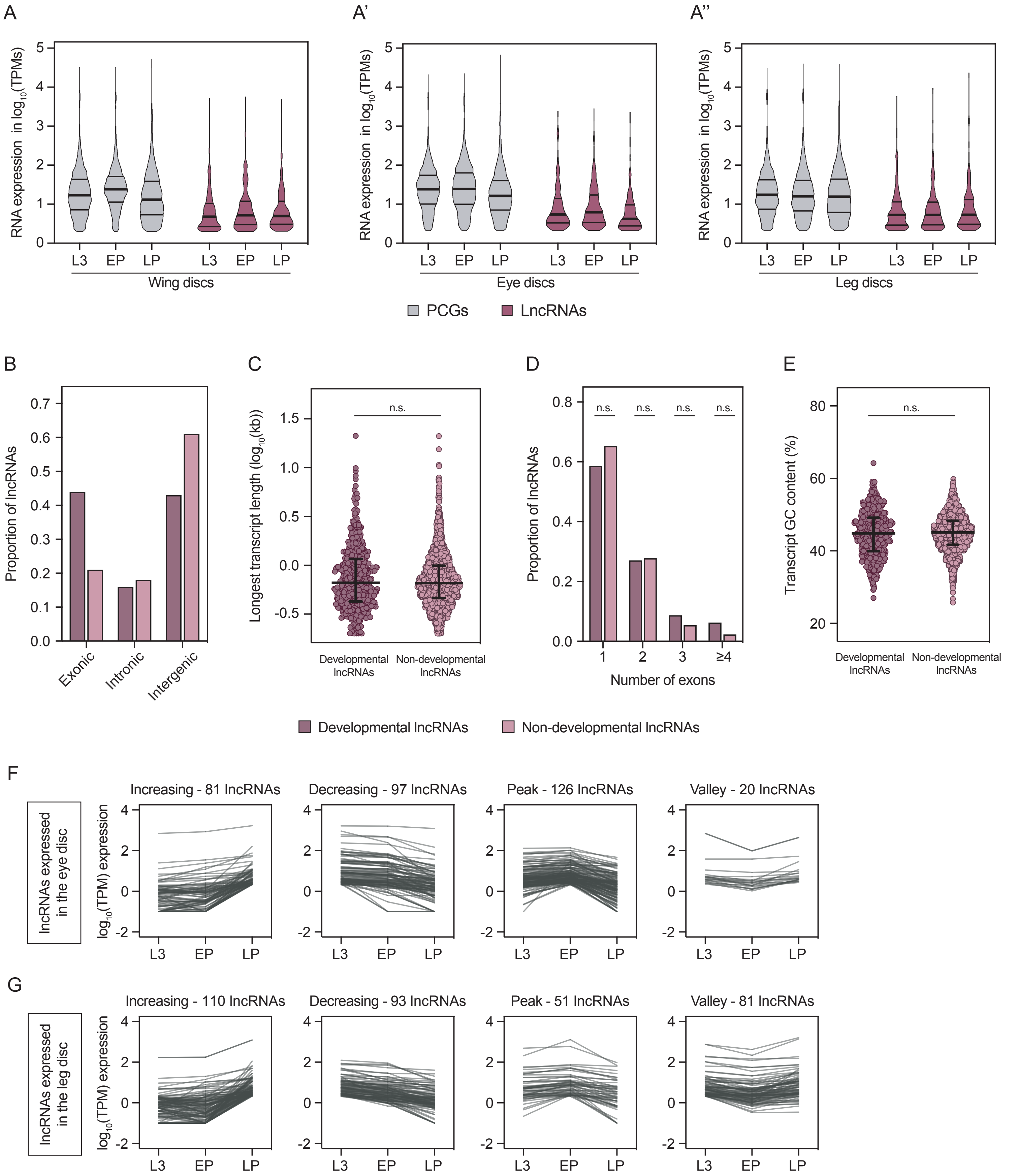

Supplemental Fig. S1. Characterization of genes expressed in developing imaginal discs. (**A-A'-A'''**) Violin plots showing the expression of PCGs and lncRNAs expressed at least 1 TPM in the wing, eye or leg discs. The first quartile, median and third quartile are represented. (**B**) Classification of developmental lncRNAs (expressed in at least one developmental sample) and non-developmental lncRNAs (not expressed in the wing, eye and leg discs) into exonic, intronic or intergenic. (**C**) Length of the longest transcript for each lncRNA. Lenght is presented as the log base 10 in kilobases. Median and interquartile range are represented. (**D**) Number of exons of the longest transcript for each lncRNA. (**E**) GC content of the longest transcript for each lncRNA. Median and interquartile range are represented. (**F**) Classification of the lncRNAs expressed in the eye disc during development. (**G**) Classification of the lncRNAs expressed in the leg disc during development. Data from Ruiz-Romero et al. 2022.

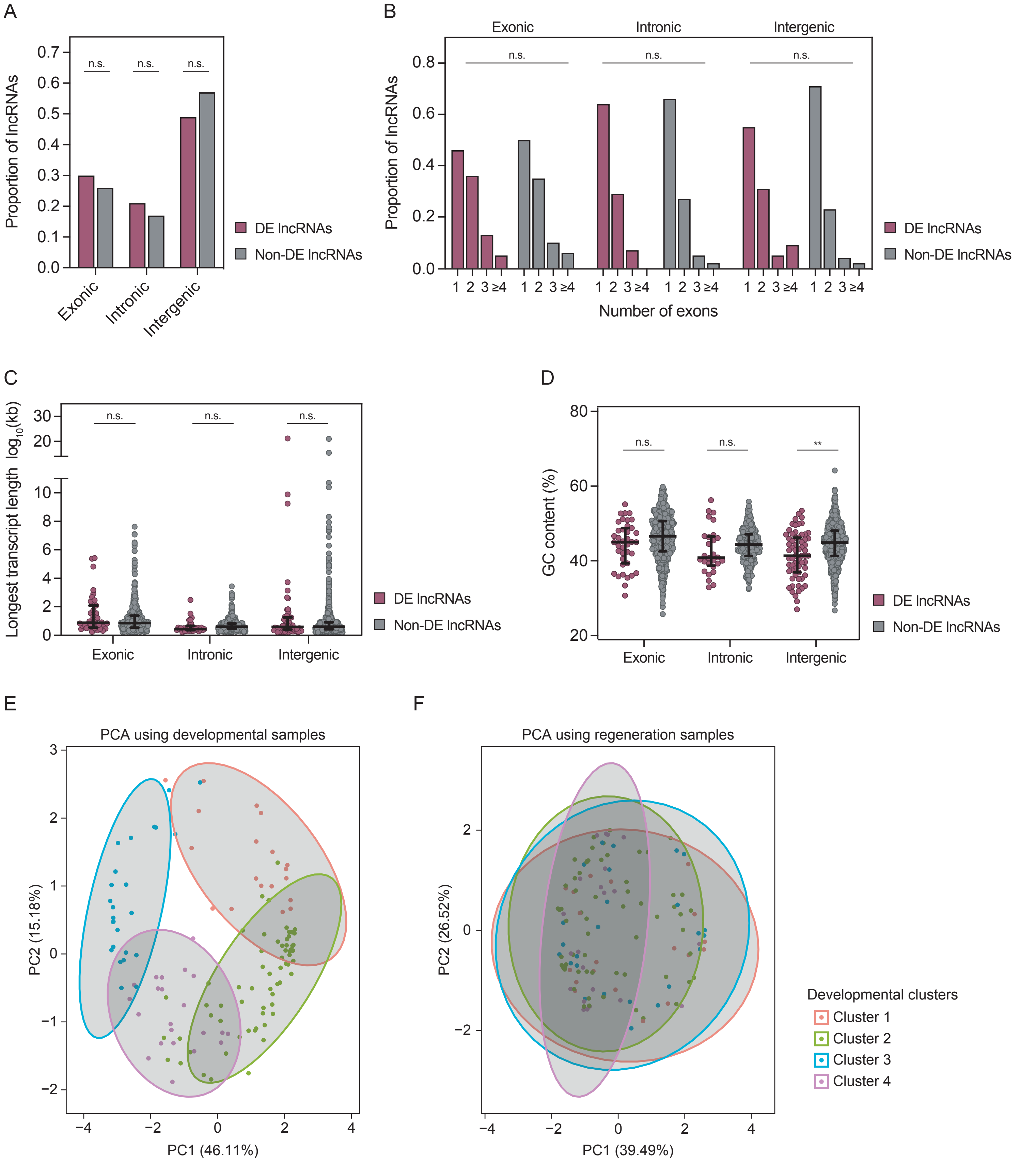

Supplemental Fig. S2. Differentially-expressed (DE) lncRNAs in regeneration. (**A**) Proportion of exonic, intronic and intergenic lncRNAs. (**B**) Classification of exonic, intronic and intergenic lncRNAs according to the number of exons in their longest transcript. (**C**) Longest transcript length of exonic, intronic and intergenic lncRNAs. (**D**) GC content of the longest transcript of exonic, intronic and intergenic lncRNAs. (**E**) Principal component analysis (PCA) based on the expression in development of DE lncRNAs. (**F**) PCA based on the expression in regeneration of DE lncRNAs. The color code in E and F represents the 4 gene clusters defined in Figure 3H. PCAs shown in E and F were based on the expression of the 129 clustered lncRNAs differentially-expressed in regeneration normalized as Z-scores. The median and interquartile range are represented in C and D. $p < 0.01$ (**); n.s. = non-significant. Data from Vizcaya-Molina et al 2018.

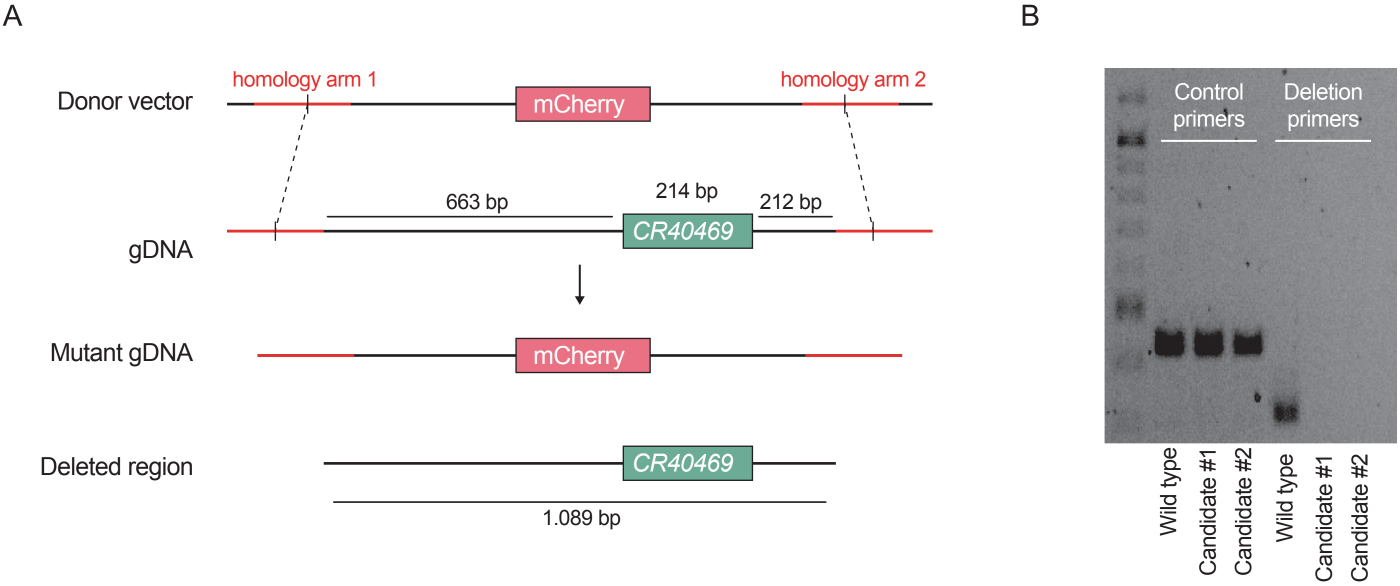

C

```
CR40469           ------------------------------------CACGTTCCTCACTAATTGTGGCTA  24
CR34335-short     GGCGGTCGAGTGCCTCACAGTGTATCAAGGGTTGGCCACGTTCCTCACTAATTGTGGCTA  60
CR34335-long      GGCGGTCGAGTGCCTCACAGTGTATCAAGGGTTGGCCACGTTCCTCACTAATTGTGGCTA  60
                                                      ************************

CR40469           TTTGCGCCATCGTCTCATGCAATGTTATTTGAGAGATGGCAAAATATATAGTATGTTTGT  84
CR34335-short     TTTGCGCCATCGTCTCATGCAATGTTATTTGAGAGATGGCAAAATATATAGTATGTTTGT  120
CR34335-long      TTTGCGCCATCGTCTCATGCAATGTTATTTGAGAGATGGCAAAATATATAGTATGTTTGT  120
                  ************************************************************

CR40469           CTCCAATGTGTTGAGACTGAGAAGATATTGTACCCGTGAATTGATGAAAATTGATTGATT  144
CR34335-short     CTCCAATGTGTTGAGACTGAGAAGATATTGTACCCGTGAATTGATGAAAATTGATTGATT  180
CR34335-long      CTCCAATGTGTTGAGACTGAGAAGATATTGTACCCGTGAATTGATGAAAATTGATTGATT  180
                  ************************************************************

CR40469           ATATTGTAATGTTGATTTCATGAAAAACACGCTGTGTTGGAGGAACTCAAACAAAACAAG  204
CR34335-short     ATAT-GTAATGTTGATTTCATGAAAAACACGCTGTGTTGGAGGAACTCAAACAAAACAAG  239
CR34335-long      ATAT-GTAATGTTGATTTCATGAAAAACACGCTGTGTTGGAGGAACTCAAACAAAACAAG  239
                  **** *******************************************************

CR40469           CAAAAAATCC--------------------------------------------------  214
CR34335-short     CATAAAATCC--------------------------------------------------  249
CR34335-long      CATAAAATCCAAAAAAAAAAAAAAAAAAACAAATCAAATTTTAACAAACAATAATAATAC  299
                  ** *******

CR40469           ------------------------------------------------------------  214
CR34335-short     ------------------------------------------------------------  249
CR34335-long      TGTGTGGTGCGCTGGCGTGGGGGAGTGGTTATTCTCACATTCCAGTCTGATCGCTTTTTT  359


CR40469           --   214
CR34335-short     --   249
CR34335-long      TT   361


Identities 212/214(99%)
Gaps 1/214(0%)
```

Supplemental Fig. S3. *CR40469* mutant validation and sequence alignment. (**A**) Schematic representation of the deleted genomic region containing the full *CR40469* locus, 663 bp upstream and 212 bp downstream. (**B**) Electrophoresis of PCR products to confirm the deletion of *CR40469* . Primers hybridizing 3 Mb downstream of the deletion site were used as control primers, while primers hybridizing within the deleted region were used to confirm the absence of the deleted sequence. The deletion was confirmed for candidates #1 and #2. (**C**) Sequence alignment of the *CR40469* and *CR34335* transcripts. The single transcript of *CR40469* and the short and long isoforms of *CR34335* were used for the alignment. 212 of the 214 nt of the *CR40469* transcript are identical in both *CR34335* isoforms. Identities are marked with an asterisk (*).

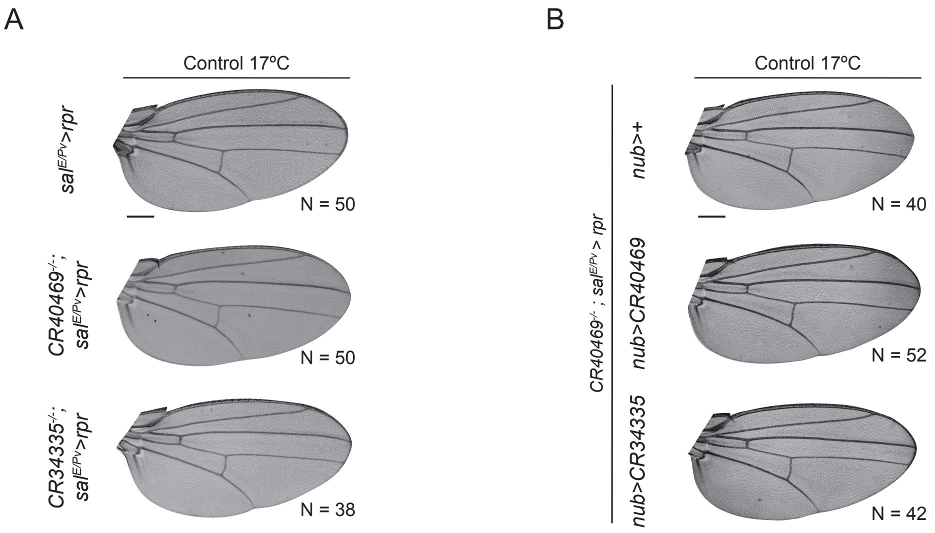

Supplemental Fig. S4. Sample images from adult wings incubated at 17ºC. (**A**) Control adult wings containing *sal*$^{E/Pv}$*>rpr* in a wild type, *CR40469* homozygous mutant, or *CR34335* homozygous mutant background. (**B**) Control adult wings containing *sal*$^{E/Pv}$*>rpr* in a *CR40469* homozygous mutant background, plus *nub*>+, *nub>CR40469* or *nub>CR34335.*

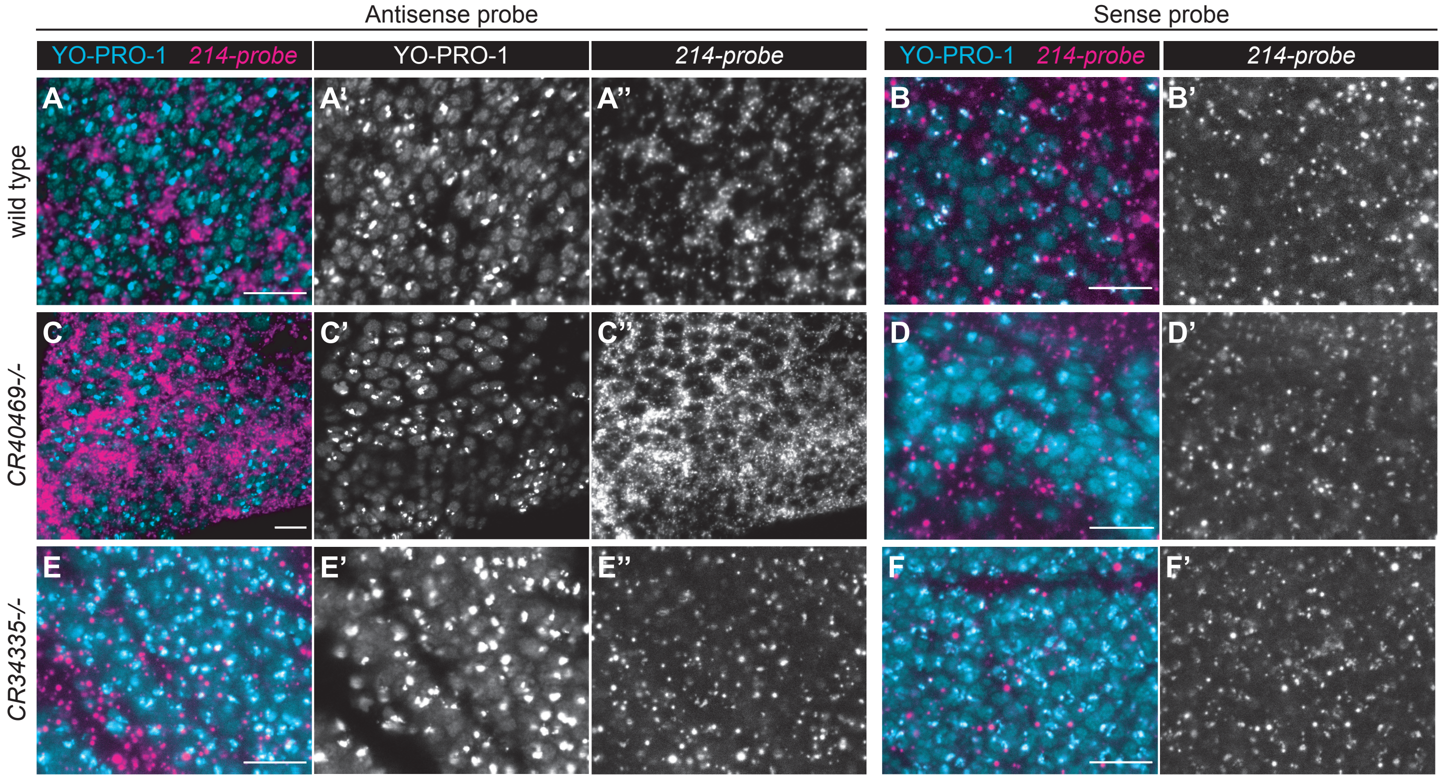

Supplemental Fig. S5. Subcellular localization of *CR40469* and *CR34335* in the wing disc. FISH imaging of (**A-B**) wild type, (**C-D**) *CR40469* homozygous mutants, and (**E-F**) *CR34335* homozygous mutants hybridized with the antisense *214-probe* designed to detect the *CR40469* and *CR34335* transcripts (A,C,E), or hybridized with a sense *214-probe* as negative control (B,D,F). N ≥ 10 per condition. Scale bar = 10 µm.

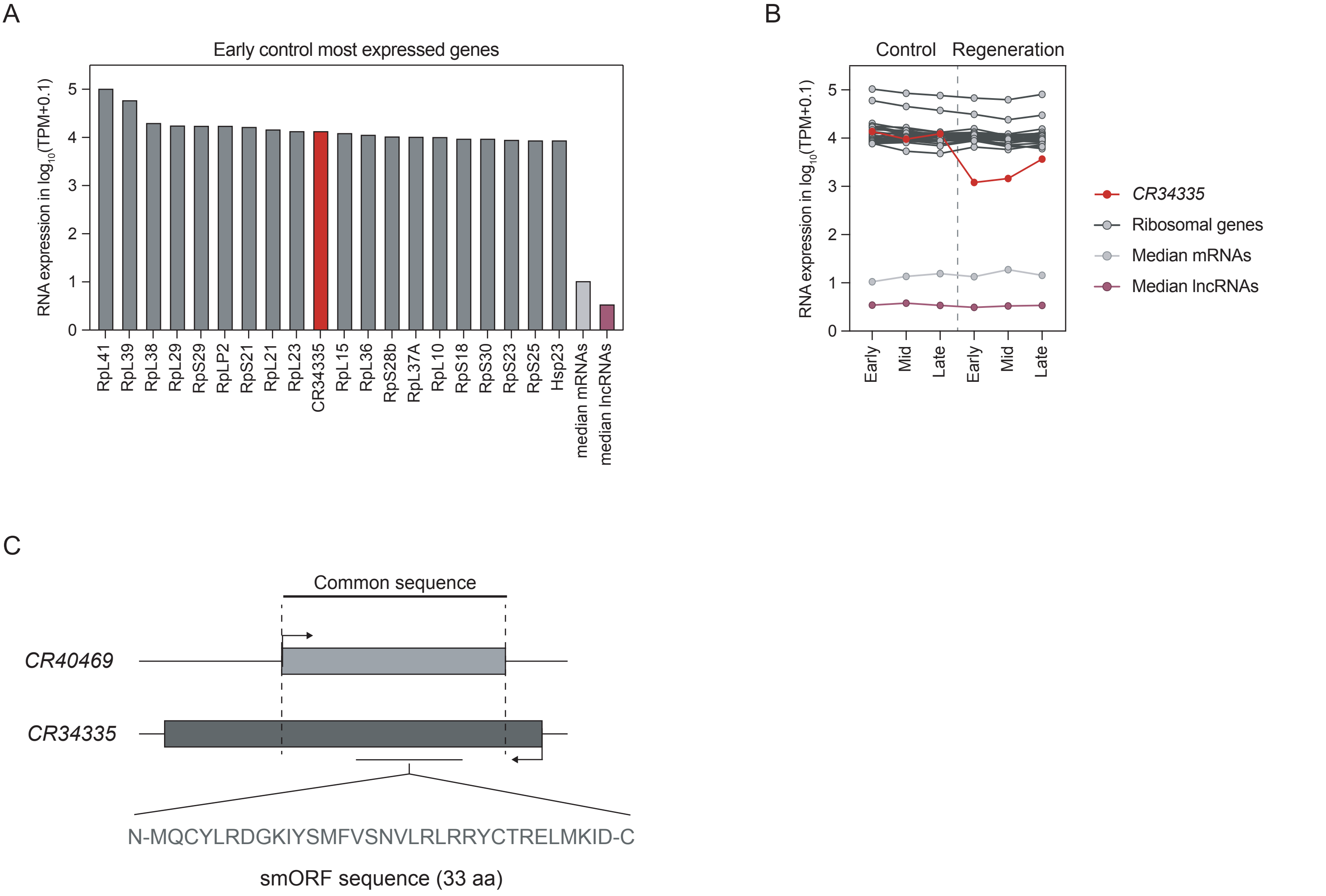

Supplemental Fig. S6. Analysis of *CR34335* expression and putative small ORF. (**A**) Expression of the 20 most expressed genes in the early control wing discs. (**B**) Expression of *CR34335* compared to that of ribosomal genes in the control and regeneration samples during the early, mid and late stages. (**C**) Predicted small ORF within the *CR40469* and *CR34335* RNA sequence using ORFinder. Localization and amino acid sequence of the predicted small ORF present in the *CR40469* and *CR34335* transcripts. Data from Vizcaya-Molina et al. 2018.

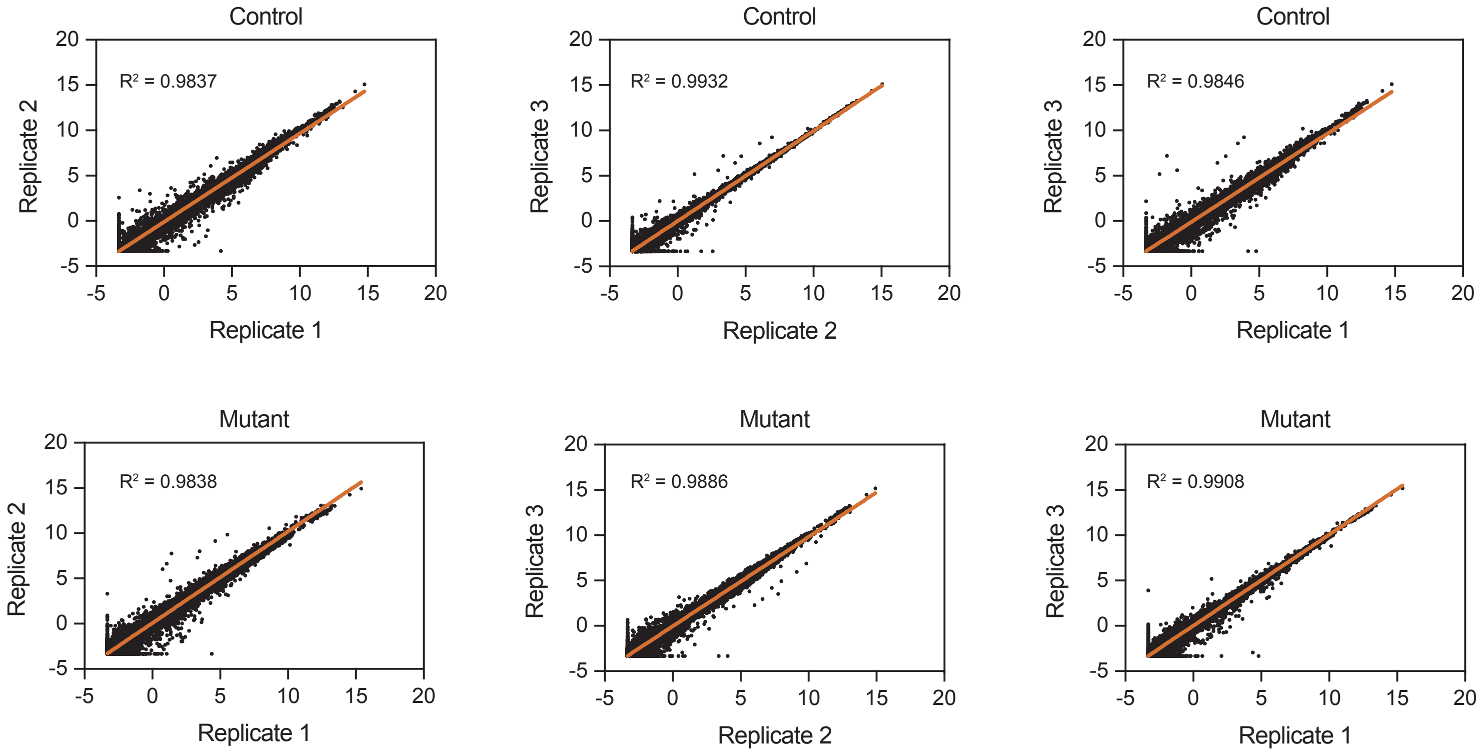

Supplemental Fig. S7. Replicate analysis of RNA-seq data. Scatter plots showing the correlation between the three biological replicates per each condition. Each dot represents the -$\log_2$ TPMs of each gene per replicate. Coefficient of determination (R^2) was higher than 0.98 in each comparison. The linear regression line is represented in orange.
